# High Throughput Multiple Locus Variable Number of Tandem Repeat Analysis (MLVA) of *Staphylococcus aureus* from Human, Animal and Food Sources

**DOI:** 10.1371/journal.pone.0033967

**Published:** 2012-05-02

**Authors:** Daniel Sobral, Stefan Schwarz, Dominique Bergonier, Anne Brisabois, Andrea T. Feßler, Florence B. Gilbert, Kristina Kadlec, Benoit Lebeau, Fabienne Loisy-Hamon, Michaël Treilles, Christine Pourcel, Gilles Vergnaud

**Affiliations:** 1 Univ Paris-Sud, Institut de Génétique et Microbiologie, UMR 8621, Orsay, France; 2 CNRS, Orsay, France; 3 Centre Européen d’Expertise et de Recherche sur les Agents Microbiens (CEERAM), La Chapelle sur Erdre, France; 4 Institute of Farm Animal Genetics, Friedrich-Loeffler-Institut (FLI), Neustadt-Mariensee, Germany; 5 INRA, UMR1225, IHAP, Toulouse, France; 6 Université de Toulouse, INP, ENVT, UMR1225, IHAP, Toulouse, France; 7 ANSES, European Union Community Reference Laboratory for Coagulase Positive Staphylococci, Maisons-Alfort, France; 8 INRA, UR1282 Infectiologie Animale et Santé Publique (IASP), Nouzilly, France; 9 Laboratoire départemental d’analyses de la Manche, Saint-Lô, France; 10 DGA/MRIS- Mission pour la Recherche et l’Innovation Scientifique, Bagneux, France; Institut Pasteur de Lille, France

## Abstract

*Staphylococcus aureus* is a major human pathogen, a relevant pathogen in veterinary medicine, and a major cause of food poisoning. Epidemiological investigation tools are needed to establish surveillance of *S. aureus* strains in humans, animals and food. In this study, we investigated 145 *S. aureus* isolates recovered from various animal species, disease conditions, food products and food poisoning events. Multiple Locus Variable Number of Tandem Repeat (VNTR) analysis (MLVA), known to be highly efficient for the genotyping of human *S. aureus* isolates, was used and shown to be equally well suited for the typing of animal *S. aureus* isolates. MLVA was improved by using sixteen VNTR loci amplified in two multiplex PCRs and analyzed by capillary electrophoresis ensuring a high throughput and high discriminatory power. The isolates were assigned to twelve known clonal complexes (CCs) and –a few singletons. Half of the test collection belonged to four CCs (CC9, CC97, CC133, CC398) previously described as mostly associated with animals. The remaining eight CCs (CC1, CC5, CC8, CC15, CC25, CC30, CC45, CC51), representing 46% of the animal isolates, are common in humans. Interestingly, isolates responsible for food poisoning show a CC distribution signature typical of human isolates and strikingly different from animal isolates, suggesting a predominantly human origin.

## Introduction

*Staphylococcus aureus* is a common commensal and frequent colonizer of humans and many animal species including companion animals as well as food-producing animals. In humans, the epithelium of the anterior nares is the primary ecological niche. *S. aureus* is also a major pathogen involved in a wide variety of diseases such as purulent skin and subcutaneous infections, pneumonia, endocarditis, abscesses and bacteremia. Moreover, *S. aureus* is an emerging issue in veterinary medicine and a cause of food poisoning by its ability to produce heat-stable enterotoxins [1].

The transfer of *S. aureus* isolates between humans and animals, especially in the case of livestock-associated MRSA ST398, has recently gained particular attention [2]. However, relatively little is known about the more global diversity of *S. aureus* isolates of animal origin [3]–[17]. This limits our ability to identify for example the origin of strains responsible for food poisoning. In order to implement control measures targeted at reservoirs and transmission routes, it is necessary to further improve current knowledge about animal-associated *S. aureus*.

Essentially three techniques are currently used for the large-scale analysis of the diversity of *S. aureus* isolates, namely multi locus sequence typing (MLST), *spa* typing, and multiple locus variable number of tandem repeats (VNTR) analysis (MLVA). In addition, pulsed field gel electrophoresis (PFGE) is still widely used and considered the gold-standard for typing *S. aureus* isolates. It has a high discriminatory power and it can be used for many bacterial pathogens. It is however not appropriate for routine interlaboratory comparisons [18]. MLST studies allowed the description of major clonal complexes (CC) underlying the *S. aureus* population structure [19], [20]. MLST suffers from its relatively high costs and has a moderate discriminatory power. The *spa* typing is a widely used method in which variations in a highly variable tandem repeat are characterized by sequencing. The Ridom Spaserver http://spaserver.ridom.de allows the designation of *spa* types [21], [22]. The *spa* typing is a very powerful tool, and is currently the most commonly used first line assay. However it may fail to identify new lineages due to inherent homoplasia and variable evolutionary rate of *spa* alleles and clustering based on *spa* data is complex. MLVA was developed more recently. Homoplasia at individual VNTR loci and potentially low variability of specific alleles are compensated at least partly by the use of multiple loci. An assay comprising as little as 8 VNTR loci (called MLVA-8_Bilthoven_ in the present report) was highly congruent with MLST and able to assign a new isolate to the correct CC for much lower costs [23]. The 8 loci were amplified in two multiplex PCRs and analyzed by capillary electrophoresis. A MLVA assay with 14 loci (MLVA-14_Orsay_) providing higher discriminatory power was used in a survey of 309 isolates including clinical MRSA isolates, nasal carriage isolates and representatives of the main CCs present in humans [24]. Both schemes can be adapted to low resolution DNA sizing equipment (such as agarose gels) as well as to higher throughput systems (such as capillary electrophoresis-based devices). MLVA data can be accessed via internet (a list of such databases is maintained on http://minisatellites.u-psud.fr). These databases can be queried even if a subset of loci is used although the discriminatory power and typing assignment precision might then be decreased.

In the present study, we have used MLVA as a first line assay, complemented when necessary by *spa* typing and MLST data. We have selected 16 loci for the MLVA assay, subsequently called MLVA-16_Orsay_, which essentially merges MLVA-8_Bilthoven_ and MLVA-14_Orsay_ and we have automated this assay. The products of two multiplex PCR amplifications were resolved by capillary electrophoresis, and the alleles from each of the 16 targeted loci were automatically identified. This expanded MLVA assay was used for the typing of 251 *S. aureus* isolates: the present retrospective investigation included 106 previously typed human clinical isolates, 98 isolates collected from various animal sources and mostly associated with a variety of diseases in these animals, 34 isolates recovered from food products, and 13 enterotoxigenic *S. aureus* from cases of food poisoning.

## Materials and Methods

### Strains

Two hundred and fifty-one isolates were included in the study. One hundred and six are human clinical isolates: ninety isolates from the HARMONY project reference collection kindly provided by Alex van Belkum were used to perform the development and initial validation of the automated MLVA protocol [25]; sixteen isolates were selected among two previously described collections to represent the diversity of clinical *S. aureus* strains from humans [24], [26]. Ninety-eight isolates were previously collected from different disease conditions in farm and domestic animals [27], [28], [29]. Thirty-four isolates were recovered from food [30]. Thirteen isolates were associated with food poisoning (Table 1 and Table S1).

**Table 1:** Origin of the animal and food isolates.

Sample origin	Animal^a^	Food^a^	Food poisoninga,b	Total^a^
Companion animals	17 (1)	NA^c^	NA^c^	17 (1)
Poultry	30 (0)	33 (33)	1(0)	64 (33)
Small ruminants	11 (0)	1(0)	7(0)	19 (0)
Swine	32 (5)	0	1(0)	33 (5)
Rodent	2 (0)	0	0	2 (0)
Horse	5 (0)	0	0	5 (0)
Cattle	1(0)	0	3 (0)	4 (0)
Total	98 (5)	34 (33)	12 (0)	144 (39)

a the number of MRSA isolates is indicated in brackets.

b the origin of one of the 13 food poisoning associated isolates is unknown.

c NA : not applicable.

### DNA Extraction

Strains were cultured overnight at 37°C in Brain Heart Infusion (BHI) or Luria Bertani broth. Genomic DNA was extracted using phenol-chloroform extraction or the DNeasy tissue kit (Qiagen, Courtaboeuf, France) after treatment with lysostaphin (100 mg/l) (Ambi products LLC, USA). Nucleic acid quality and concentration were estimated with an ND-1000 spectrophotometer (NanoDrop, Labtech, Palaiseau, France). Samples diluted in water at 5 ng/µl were used as DNA template for PCR amplification.

### Selection of VNTRs and MLVA Typing

Twelve loci previously investigated by Pourcel *et al.* and all eight loci used by Schouls *et al.* [23], [24] were merged in a single assay comprising 16 loci. The 16 VNTR loci were amplified in two multiplex PCRs using the ceeramTools® *Staphylococcus* typing kit (Ceeram, La Chapelle sur Erdre, France). PCR reaction 1 amplifies the ten VNTR loci Sa0122, Sa0311, Sa0387, Sa0550, Sa0684, Sa0964, Sa1097, Sa1194, Sa1729, Sa1866. PCR reaction 2 amplifies the six VNTR loci Sa0266, Sa0704, Sa1132, Sa1291, Sa2039, Sa2511 (Tables 2 and 3). VNTRs Sa0122 and Sa0266 are located in the genes *spa* and *coa* respectively. VNTRs Sa0311, Sa1729 and Sa1866 are members of the family of intergenic repeated elements called “STAR” for *S. aureus* repeats [31].

**Table 2:** Constitution of the different MLVA schemes.

VNTR^a^	Unit size	Alias^b^	MLVA-16_Orsay_	MVLVA-14_Orsay_ g	MLVA-10_Orsay_ h	MLVA-8_Bilthoven_ i
Sa0122	24	Spa, SIRU21^c^, VNTR24_01^d^	X	X	X	X
Sa0266	81	Coa, VNTR81_01^d^	X	X	X	X
Sa0311	55		X	X	X	
Sa0387	55	SIRU1^c^	X			
Sa0550	21	VNTR21_01^d^	X			X
Sa0684	61	VNTR61_02^d^	X			X
Sa0704	67	VNTR67_01^d^	X	X	X	X
Sa0906	56			X		
Sa0964	43	SAV0920-0921^e^	X			
Sa1097	9	Sspa^f^, VNTR09_01^d^	X			X
Sa1132	63	VNTR63_01^d^	X	X	X	X
Sa1194	67		X	X	X	
Sa1213	56			X		
Sa1291	64	SIRU13^c^	X	X	X	
Sa1425	58			X		
Sa1729	56		X	X	X	
Sa1756	131	SIRU15^c^		X		
Sa1866	129		X	X	X	
Sa2039	56		X	X	X	
Sa2511	61	VNTR61_01^d^	X			X

a the VNTR locus name reflects genome localisation (in kb) in strain Mu50 refseq NC_002758.

b alternative names given in the literature.

c from [52].

d from [23].

e from [53].

f from [54].

g MLVA14_Orsay_ corresponds to the 14 panel 1-panel 2 loci in [24].

h MLVA10_Orsay_ corresponds to the 10 panel 1 loci in [24].

i MLVA8_Bilthoven_ corresponds to the 8 loci used by [23].

**Table 3:** VNTRs and associated oligonucleotide primers.

VNTR name	PCR^a^	Forward primer sequence (5′ → 3′)	Reverse primer sequence (5′ → 3′)	Unit size (bp)	Mu50 size^b^	Allele size range^c^	HGDI* [95% confidence interval]
Sa0122	1	NED-CAGCAGTAGTGCCGTTTG	GCACCAAAAGAGGAAGAC	24	331 (10)	115–475 (1–16)	0.80 [0.759,0.847]
Sa0266	2	PET-TTGGATATGAAGCGAGACCA	CTTCCGATTGTTCGATGCTT	81	630 (6)	387–954 (3–10)	0.67 [0.632,0.705]
Sa0311	1	VIC-GTATCAACAAGTGATAGCATCA	AAATGATATTTTCGCAAAATTTATT	55	316 (3)	206–426 (1–5)	0.64 [0.585,0.691]
Sa0387	1	6FAM-CAAAGTAATAGGCACTACAA	CATTCCAAACATACCATCAC	55	255 (2)	179–420 (0.5–5)	0.73 [0.685,0.767]
Sa0550	1	PET-GTGACAGATGTAAGACTTAGA	AACTTGATCGACACCAGAGC	21	847 (5)	784–847,1183–1246 (2–5,21–24)	0.37 [0.257,0.490]
Sa0684	1	6FAM-AGGTATTGGAAGTGAAACAGC	CAACAAGTTGTTCAGCCTGC	61	1000 (2)	939–1183 (1–5)	0.52 [0.420,0.616]
Sa0704	2	VIC-AAGAGTGTGTAGGGAATGGC	CGATCGCACGATATGGTGG	67	307 (4)	173–709 (2–10)	0.76 [0.710,0.801]
Sa0964	1	PET-ATCCCAGATTATCCAATACAA	CCAACCTGTTAATCCGATGT	43	597 (6)	382–769 (1–10)	0.61 [0.539,0.676]
Sa1097	1	PET-GAATTATTGTTATCGCCATTGTC	GCAACTTCTTAAAACAAAATATTG	9	196 (15)	124–241, 286 (7–20,25)	0.65 [0.558,0.751]
Sa1132	2	6FAM-CTAGTTCAAGCTAGATCAGG	TGGGAGGAATTAATCATGTC	63	884 (7)	569–1262 (2–13)	0.67 [0.611,0.720]
Sa1194	1	NED-CTGTGTCGGTAGGTTACATT	GGTGCTAAAGTCGATGTAAT	67	874 (7)	539–1008 (2–9)	0.59 [0.506,0.673]
Sa1291	2	NED-GTCAAGACACAGATATTGCT	GTGTTGCTCTTGAATCATC	64	870 (4)	678–1254 (1–10)	0.55 [0.442,0.657]
Sa1729	1	6FAM-GTCTCGAATCACTTAACAACG	GACCATGCACTACGTGTTAC	56	797 (5)	573–853 (1–6)	0.59 [0.522,0.669]
Sa1866	1	VIC-GCTTTACGTGTAATAACACC	GCTTGTTGGTTCAGCTTTAGG	159	933 (3)	522–1092 (0.5–4)	0.49 [0.400,0.587]
Sa2039	2	6FAM-TATTTCGTTCTACCCCAACT	CATAAATTCAATGTCCTAGGC	56	275 (3)	163–443 (1–6)	0.65 [0.565,0.726]
Sa2511	2	NED-GGCAAAATGCACATGAAACACT	AAGTCAAGAATATTTAAAATCAATT	61	370 (3)	248–675 (1–8)	0.82 [0.781,0.860]

(*HGDI calculated on the 90 isolates from the HARMONY collection).

a Multiplex PCR reaction.

b Expected amplicon size for strain Mu50 RefSeq NC_002758 (by convention, corresponding number of repeated units for strain Mu50).

c Observed allele size range: amplicons length (number of repeated units).

Briefly, the kit includes two primer mixes, one for each multiplex reaction. Forward primers were fluorescently labeled at the 5′ end, reverse primers were synthesized unlabeled and tailed (Applied Biosystems, Courtaboeuf, France) as previously described [32]. Both multiplex PCRs were performed in a final volume of 15 µl using the Qiagen multiplex PCR kit (Qiagen, Courtaboeuf, France). The reactions contained 2 µl template DNA (5 ng/µl), 7.5 µl of 2× multiplex PCR mastermix and 5.5 µl of primer mix. The PCR reactions were run on a Veriti® thermal cycler (Applied Biosystems, Courtaboeuf, France) using the following conditions: initial denaturation cycle for 15 min at 95°C, 15 cycles touchdown PCR (30 s at 95°C; 60 s at 75°C, with 1.0°C drop in temperature each next cycle, 70 s at 72°C), 15 cycles long range PCR (30 s at 95°C, 60 s at 60°C, 70 s at 72°C with 5 s increase in time each next cycle), with a final 10 min step at 72°C. PCR fragments were purified using Qiagen DyeEx plates (Qiagen, Courtaboeuf, France). For each multiplex reaction, 2 µl of purified PCR product were combined with 7.75 µl HiDi formamide and 0.25 µl GS1200LIZ (Applied Biosystems, Courtaboeuf, France). Samples were loaded onto the ABI3130 capillary sequencer using a 50 cm capillary filled with performance-optimized polymer 7 (Applied Biosystems, Courtaboeuf, France) at 60°C for 6200 s with a running voltage of 12 kV, and an injection time of 10 s at an injection voltage of 1.6 kV.

### MLVA Data Analysis

**Figure 1:**
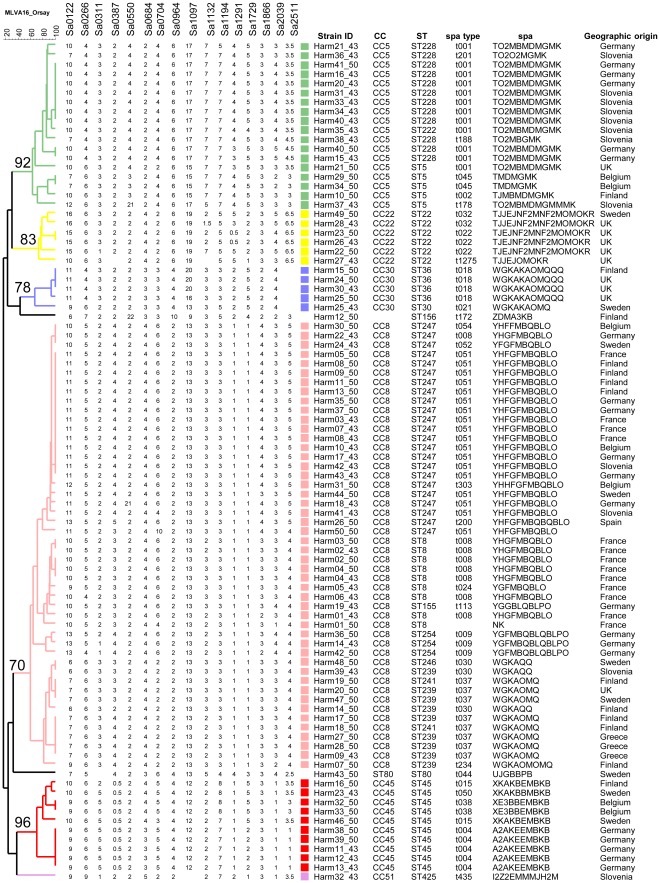
Dendrogram of the HARMONY collection using MLVA-16_Orsay_. Color coding is according to MLST clonal complex assignment whereas clustering is done according to the displayed MLVA data. Strain Id, clonal complex, sequence type, spa type, spa code and geographic origin are indicated. MLVA cluster bootstrap values are shown for the main clusters.

Each VNTR locus was identified according to color and automatically assigned a size by the GeneMapper software (Applied Biosystems, Courtaboeuf, France). This size was then converted into an allele designation associated with a quality index. Rare intermediate-sized alleles were reported as half-size (.5, Figure 1 and Figure S1) as previously described [24]. New alleles of unexpected size were sequenced. The typing data file was imported into BioNumerics version 6.6 (Applied-Maths, Sint-Martens-Latem, Belgium). Allele designations and allele calling conventions for the VNTRs described in previous work were used as published [24]. The MLVA code is provided in the order corresponding to the genome position in reference strain Mu50 (refseq accession number NC_002758): Sa0122, Sa0266, Sa0311, Sa0387, Sa0550, Sa0684, Sa0704, Sa0964, Sa1097, Sa1132, Sa1194, Sa1291, Sa1729, Sa1866, Sa2039, Sa2511 [33]. Following these conventions, the genotype of the reference strain Mu50 deduced from its genomic sequence is 10-6-3-2-5-2-4-6-15-7-7-4-5-3-3-3 (Table 3).

The diversity (*D*) index and confidence intervals (CI) were calculated as previously described [32]. The UPGMA (unweighted pair group method with arithmetic mean) clustering method was run using the categorical distance coefficient. A cut-off value of 45% similarity was applied to define clusters [24].

### Analysis of Linkage Disequilibrium, Bootstrapping and Congruence Between Different Methods

Linkage disequilibrium was measured by using LIAN Ver. 3.5 software accessed at http://guanine.evolbio.mpg.de/ [34]. The Monte-Carlo simulation was run with 100000 iterations. Bootstrap analyses were run with 500 simulations.

### Comparison of Animal, Human and Food Poisoning Isolates

Fischer’s exact test was applied to compare the proportions of human-related CCs in three different populations: isolates from animals, isolates from humans and isolates involved in food poisoning. Data from the literature [3]–[9], [17] and from the present study were analysed to build the population of animal-associated isolates. We combined our results with those described by Wattinger *et al.* including 20 isolates from food poisoning events, to form the population of 33 isolates involved in food poisoning [35].

### DNA Sequencing

PCR amplicons were purified using the QIAquick PCR purification (Qiagen, Courtaboeuf, France) and sequenced (Cogenics, UK or Eurofins MWG Operon, Ebersberg, Germany). Sequence data were managed with BioNumerics. The primers and conditions used for the MLST or *spa* tandem repeat amplification were as described by Enright *et al.* and Harmsen *et al.* respectively [21], [22]. Alleles and sequence types (STs) were identified using the MLST database (http://www.mlst.net). The *spa* repeat nomenclature was that of Shopsin *et al*., and *spa* types were retrieved from the Ridom SpaServer http://spaserver.ridom.de [36].

## Results

### Automated Multiplex Capillary-based MLVA Assay Development

**Figure 2:**
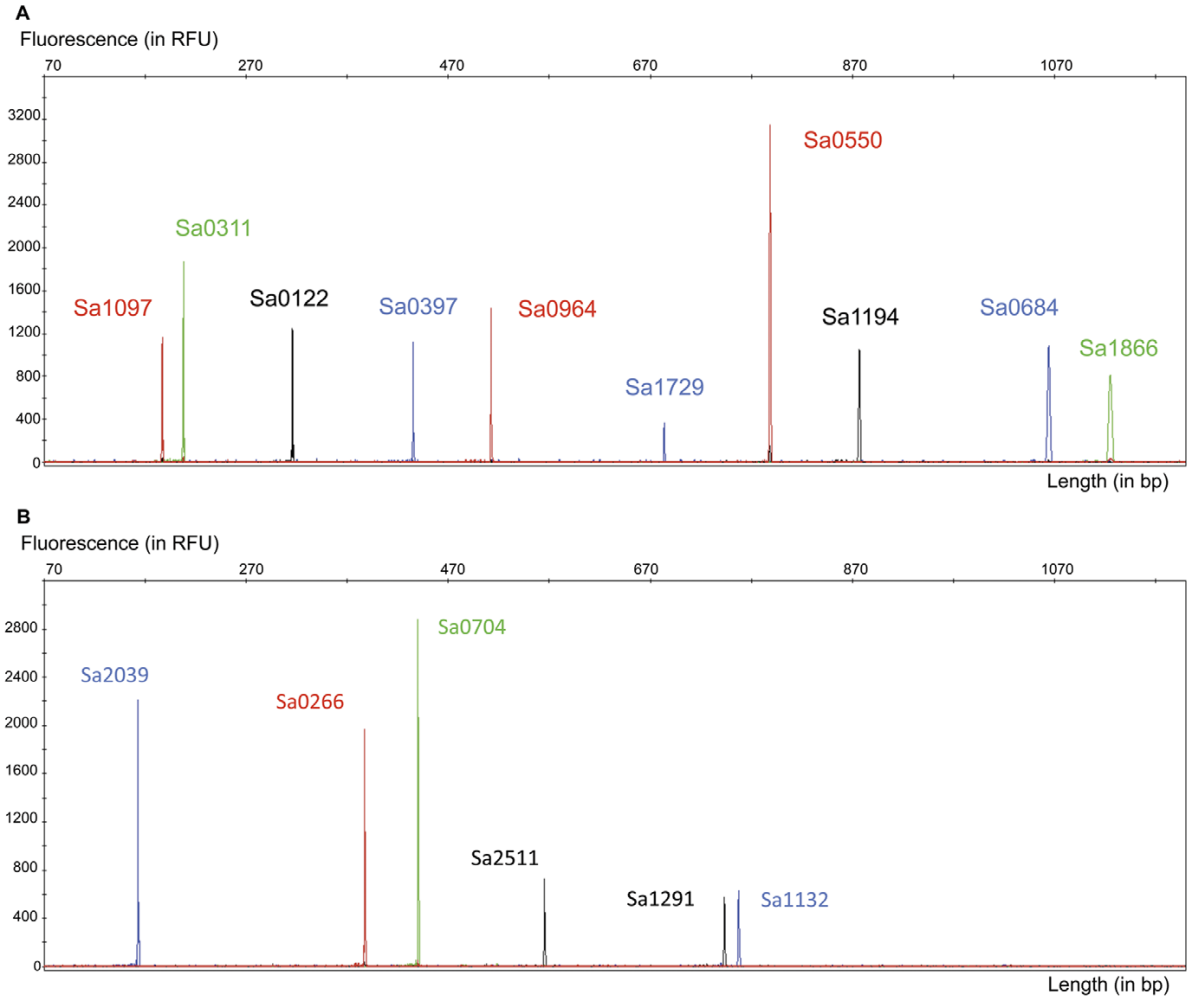
Electrophoregrams showing multiplex PCR amplicons resolved by capillary electrophoresis **a. PCR1** ten dye-colored coamplified VNTR loci. **b. PCR2** six dye-colored coamplified VNTR loci.

MLVA-16_Orsay_ integrates the two most recently published MLVA assays, each associated with large databases accessible via internet [23], [24] (Table 2). The resulting data can be compared to both data sets. Figure 2 shows a typical capillary electrophoresis pattern of the two multiplex PCRs (Figure 2a PCR1, 10 loci; Figure 2b, PCR2, 6 loci). It preserves the convenient 2-multiplex PCR assay developed by Schouls *et al.* while including 8 additional VNTR loci [23].

### Efficiency of the MLVA-16_Orsay_ Protocol

The MLVA-16_Orsay_ scheme was first tested on 90 well-described isolates of the HARMONY reference collection (Figure 1). These isolates represent epidemic or major nosocomial MRSA clones from the mid-1980s to 1998 and were previously investigated by MLVA-14_Orsay_ [24]. Nine missing values were observed in the present study among the expected 1440 values. Sa2511 was not amplified in five closely related isolates. Thus, MLVA-16_Orsay_ provided high typeability (T value = 99.4%). The discriminatory index *D* was 0.9625, compared to 0.9531 for subset MLVA-8_Bilthoven_. Forty-nine types were identified when using the 16 loci, compared to 41 with the 8 loci assay.

### Congruence Between MLVA-16_Orsay_ and MLST

**Figure 3:**
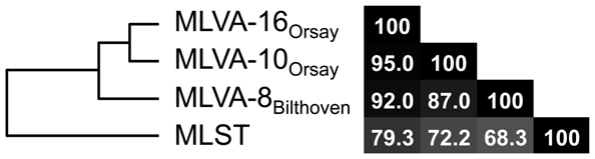
Congruence of MLVA and MLST. Congruence of MLVA schemes (MLVA-16_Orsay,_ MLVA-10_Orsay_, and MLVA-8_Bilthoven_) and MLST using a Pearson correlation coefficient.

Direct comparison between MLST and MLVA clustering based on the 90 isolates of the HARMONY collection showed a strong correlation between these two genotyping methods (Figure 1 and Figure 3), in agreement with previous reports [23], [24]. For instance, the congruence between MLST and MLVA-16_Orsay_ was 79.3% and MLVA correctly assigned isolates to their MLST defined CC. CC nodes were supported by high bootstrap values demonstrating the reliability of MLVA-16_Orsay_ clustering for *S. aureus* population investigation (Figure 1). MLVA-16_Orsay_ was much more discriminatory than MLST (MLST distinguishes 17 STs compared to the 49 MLVA**-**16_Orsay_ types and the discriminatory index *D* for MLST is 0.8856). The standardized index of linkage association for MLST was 0.349. In comparison the standardized index of linkage association for MLVA-16_Orsay_ was 0.242. The detected linkage disequilibrium was highly significant in both cases (P<10^−5^). The lower linkage detected by MLVA was previously observed in *Legionella pneumophila* and might be a consequence of homoplasy at VNTR loci [37].

### Diversity Among *S. aureus* Isolates Collected from Animals

**Figure 4:**
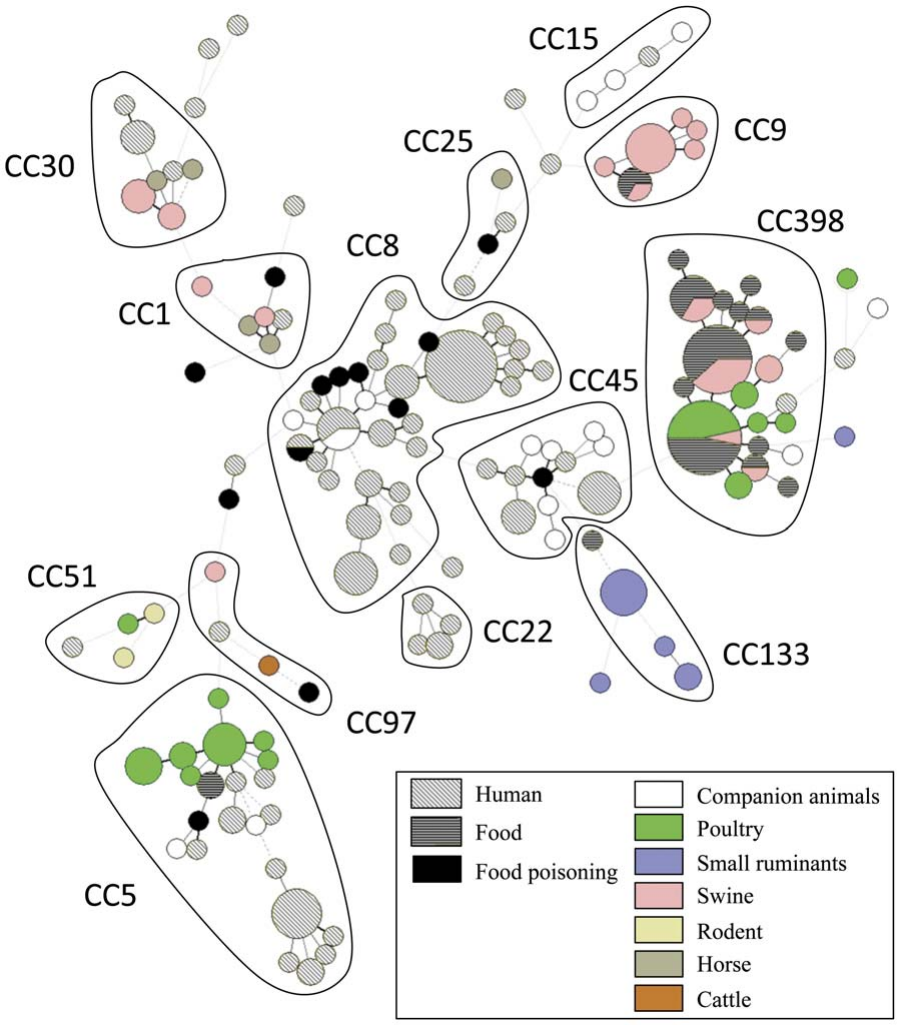
Minimum spanning tree of the 251 *S. aureus* isolates using MLVA-16_Orsay_. Minimum spanning tree of the 251 *S. aureus* isolates (106 human-associated isolates, 98 animal-associated isolates and 47 isolates from food products among which 13 were related with food-poisoning) using MLVA-16_Orsay_. Each circle represents a MLVA genotype. The size of each circle indicates the number of isolates within this MLVA genotype. The different clusters are annotated. The host origin is indicated with a specific color. Isolates involved in food poisoning events are represented by black circles. Human and food isolates are highlighted with two different hatch patterns.

The MLVA-16_Orsay_ assay was used to type 98 animal isolates. A full data set was obtained with one exception: VNTR Sa1291 could not be amplified in isolate sa263. The 98 isolates were resolved into 59 MLVA-16_Orsay_ types (MTs) distributed in 12 clusters and 4 singletons as shown on the minimum spanning tree of Figure 4. MLVA-8_Bilthoven_ resolved 47 MTs (*D* = 0.9571). In terms of diversity, human-associated (106 isolates) and animal-associated (98 isolates) isolates were similar, with MLVA-16_Orsay_ *D* values of 0.9727 and 0.9788, respectively.

### CC Distribution of the Animal-associated Isolates Deduced from Previously Published or *de novo* MLST Data

The tentative identification of the clusters in the animal and food-product isolates was done by comparison with previously published data [23], [24], [26]. The *spa* typing was used to confirm some of the assignments. The largest clusters were CC398 and CC5, which comprised 27% (26 isolates) and 17% (17 isolates) of the typed isolates, respectively (Figure S1). Eleven MLVA-16_Orsay_ genotypes were observed in CC398. Three MLVA-16_Orsay_ clusters comprising 12, 9 and 2 isolates could be assigned to CC9, CC133 and CC97 respectively (Figure S1). Figure 4 shows the distribution of animal strains among the different CCs. The 32 porcine isolates clustered into 5 CCs, CC398 (12 isolates, 38%), CC9 (12 isolates, 38%), CC30 (5 isolates, 15%), CC1 (2 isolates, 6%) and CC97 (1 isolate, 3%). All porcine MRSA isolates belonged to CC398. The 30 isolates from poultry were exclusively MSSA and clustered mainly in 2 CCs: CC398 (13 isolates, 43%) and CC5 (15 isolates, 50%). The 17 isolates from dogs and cats were all MSSA, except for one MRSA CC398 isolate. They were distributed into 4 CCs, CC45 (6 isolates, 35.3%), CC8 (4 isolates, 23.5%), CC15 (3 isolates, 17.6%), CC5 (2 isolates) and a singleton (sa263). The 11 isolates from small ruminants comprised nine CC133 isolates (these MSSA isolates were obtained from sheep of the same German flock suggesting the presence of an epidemic strain) and two non-grouped isolates from mastitis. The five isolates from horses, all MSSA, belonged to three clusters, CC1 (two isolates), CC30 (two isolates) and CC25 (one isolate). Notably, both CC1 isolates shared the same MLVA-16_Orsay_ profile although they were collected in two different countries and 16 years apart. Similarly, the two rodent isolates belonged to the same rare lineage (CC51) although they were collected in two different places and from different disease conditions.

### CC Distribution Among the Food-associated Isolates of the Test Collection

Two groups of isolates were recovered from food: 13 isolates originated from cases of food-poisoning and 34 MRSA isolates originated from food not related to poisoning events (food isolates are exclusively MRSA because of the screening procedure [30]). Seven of the 13 food-poisoning associated isolates were collected from dairy products, five were recovered from meat products, and the precise food origin of one isolate was unknown (Figure 4, Table 1 and Table S1). Altogether, 6 isolates belonged to CC8, and single isolates to CC1, CC5, CC25, CC45, CC97, two isolates were not assigned to CCs. The second group was almost exclusively composed of CC398 isolates from poultry meat or poultry meat products (29 of 34 isolates). The remaining five isolates belonged to CC5, CC9 or CC133. Figure S2 shows a minimum spanning tree of all 55 CC398 isolates from animal or food investigated. Figure S3 shows a minimum spanning tree of all isolates based upon MLVA-16_Orsay_ data. Animal and food samples are colored (green for MSSA samples, red for MRSA samples, poultry samples are cross-hatched).

### Meta-analysis of Human-related *S. aureus* CCs Prevalence in Food Products Involved in Staphylococcal Intoxications

We searched the literature for reports investigating *S. aureus* in animals. We identified 836 farm animal isolates for which a CC assignment is known [3], [4], [5], [6], [7], [8], [9], [17]. Ten (1.2%) and four (0.5%) belonged to CC8 and CC45, respectively. In contrast CC8 and CC45 represent 10–40% and 10–20% of human isolates respectively [24], [26], [38], [39], [40]. Thirty percent (10/33) and twenty one percent (7/33) of the thirty-three isolates involved in food poisoning investigated in this study or by Wattinger *et al.* [35], belonged to CC8 and CC45 respectively. The difference between the proportion of CC8/CC45 in the animal-associated isolates population and the food poisoning isolates is highly significant according to Fisher’s exact test (P<0.05). In sharp contrast, the proportion of CC8/CC45 among food poisoning isolates is highly similar to the proportion of CC8/CC45 isolates among human isolates. This observation strongly suggested that the isolates associated with food poisoning were mainly of human origin.

### High Discriminatory Power of MLVA-16_Orsay_ in CC398

MLVA-16_Orsay_ distinguished 19 genotypes with a diversity index of 0.8728 (Figure S2 part A). In comparison, 9 genotypes are resolved when using the MLVA-8_Bilthoven_ subset, with a much lower diversity index of 0.6728 (Figure S2 part B). This difference is largely due to locus Sa1291 (Figure S1) which is not otherwise an exceptionally variable locus (Table 3).

## Discussion

The trace-back analysis of food-borne infections requires the availability of appropriate genotyping tools, *i.e.* highly cost-efficient and fast methods for high-throughput analysis, backed up by relevant and easily accessible typing databases. The present investigation further illustrates the relevance of MLVA for large scale population investigations of *S. aureus*, a major pathogen and source of food intoxications.

### Automated Capillary-based 16 loci MLVA Assay

In the commercially available typing kit (ceeramTools®, Ceeram, La Chapelle sur Erdre, France), 16 loci are amplified in two multiplex PCRs. These 16 loci were chosen in order to provide data directly comparable with previously developed databases based upon the typing of 2 subsets of these loci [23], [24]. The assay is able to correctly assign *S. aureus* isolates to defined MLST clonal complexes and further differentiate within these CCs. Homoplasy associated with VNTR loci is presumably efficiently compensated by the analysis of multiple loci. MLVA is equally well adapted for studying *S. aureus* epidemiology regardless of the sample origin (animal or human). The four steps of the MLVA procedure (DNA extraction, amplification of the 16 VNTRs in 2 multiplex PCRs, fragment analysis by capillary electrophoresis, MLVA code assignment) were standardized to be usable and understandable by non-expert users. The resulting data can be queried against freely accessible internet MLVA databases such as http://mlva.u-psud.fr.

### MLVA-based Diversity Analysis of Animal-associated *S. aureus*

Ninety-six percent of the isolates were clustered within twelve known CCs. Based on MLVA results, the studied animal-associated isolates were globally as diverse as human-associated ones suggesting that, as a rule, the occurrence of *S. aureus* among animals is not a recent event.

Approximately half of the animal isolates belonged to eight well-known human CCs in agreement with previous studies showing the wide host range of some CCs [3], [4], [5]. However, recent host adaptation, sporadic contamination or the presence of widespread lineages must also be taken into account as possible explanations. Evidence for recent host adaptation is provided by the well-described CC5 isolates in poultry [41]. In the present study, companion animal-associated isolates are almost exclusively found in three main clinical-associated CCs (CC8, CC15 and CC45). This illustrates the spread of *S. aureus* isolates from humans to animals [42].

### Animal-adapted Clonal Complexes

In contrast, the other half of the animal isolates is assigned to CCs not found or uncommon in humans (CC9, CC97, CC133 and CC398) [16]. This observation confirms previous studies suggesting the existence of animal-specific lineages [4], [5], [6], [10], [13], [14], [43]. Recently, Guinane *et al*. have provided evidence that CC133 which is a frequent colonizer of small ruminants, evolved as a host switch from human to ruminant followed by adaptive genome diversification [43]. MSSA-CC9 isolates have been reported in pig farmers and also from infections of swine in France and Germany showing that CC9 is able to change hosts and colonize humans [44], [45]. Two MRSA-CC9 from chicken meat were identified [30]. This lineage is very uncommon and recently emerged from its porcine reservoir in Asia [46], [47], [48], [49]. Clinical human cases due to MRSA-CC9 appeared in the same period [48].

### The Emerging CC398

In this study, CC398 was found equally in isolates collected from pigs or poultry. Half of the poultry-associated isolates belonged to CC398 whereas CC5, rather than CC398, was previously shown to be the dominant lineage in poultry. This is, to our knowledge, the second report of MSSA CC398 isolates from poultry. This observation might suggest that the lineage was already present among poultry as MSSA and has subsequently evolved as MRSA by independent acquisition of different SCC*mec* elements. MSSA CC398 could have disseminated from pigs to other food-producing animals, perhaps via farm workers, and the SCC*mec* cassette could have been acquired in other hosts. Alternatively strains may spontaneously excise part or all of the SCC*mec* and thus reverse to MSSA [50], [51]. Within CC398, various closely related spa types have been described (i.e. t011, t034, t108, t539 and t1793). Schouls and colleagues investigated 216 isolates belonging to CC398, among which 100 were pig-related [23]. They observed little diversity within this complex using MLVA-8_Bilthoven_ (*D* = 0.721). A similar results was obtained here with MLVA-8_Bilthoven_ (9 genotypes in 55 isolates, *D* = 0.6728). In contrast, the 16-loci MLVA assay discriminates 19 genotypes (*D* = 0.8728), suggesting that it might be of high interest to further differentiate CC398 isolates. Due to the higher multiplexing achieved in the MLVA-16_Orsay_ assay as described here compared to MLVA-8_Bilthoven_, the typing of 16 loci instead of 8 does not significantly increase the cost and workload.

### MLVA Typing as A Microbial Source Tracking Tool

The present investigation suggested that *S. aureus* isolates involved in food poisoning are mainly strains found in humans rather than in animals. Among the 33 isolates sampled from food products involved in food poisoning and investigated in this study and by Wattinger *et al.* [35], 10 and 7 belonged to the predominantly human clusters CC8 and CC45, respectively. Given the high human host specificity of CC8 and CC45, this finding provided evidence for the role of humans as a major source of contamination. In sharp contrast, food isolates not associated with food poisoning were almost exclusively assigned to animal-specific clones (CC398, CC9 and CC133) and no CC8 or CC45 isolates were found. In the present study, the CC97 isolate 363F is the only isolate implicated in a food intoxication event with a most likely animal origin. Although these observations need to be substantiated by the analysis of a larger test population, they point towards the role of humans in the contamination of food with enterotoxigenic *S. aureus*.

In conclusion, we merged in this work previous MLVA schemes in a rapid and efficient automated multiplex capillary-based MLVA assay for the high-resolution genotyping of *S. aureus* isolates. The numeric MLVA code is produced automatically and a quality score can be defined facilitating the development of quality controlled databases. The described MLVA-16_Orsay_ assay ensures the same clustering as MLST, assigning similarly *S. aureus* isolates to MLST defined clonal complexes. *S. aureus* MLVA typing data from the 16 loci or any convenient subset can be queried via http://mlva.u-psud.fr. MLVA is well-suited and compatible for genotyping of animal-associated *S. aureus* as well as human isolates. The present molecular typing analysis provided further insights into the diversity of animal-associated *S. aureus*. We highlighted that the animal-associated population is very diverse suggesting that animal colonization by *S. aureus* is globally ancient. In the present study, the *S. aureus* isolates from animals were divided into human-related and animal-specific CCs. Some CCs are able to switch between a great variety of hosts (*i.e.* CC5, CC45), whereas others seem to be strongly specific to particular human or animal hosts (*i.e.* CC9, CC133, CC8). The presented data indicates that *S. aureus* isolates from cases of food poisoning were most likely of human origin.

## Supporting Information

Figure S1 **Dendrogram deduced from the clustering of the 251** ***S. aureus*** **animal-associated isolates and human strains from the HARMONY collection using MLVA-16_Orsay_.** The color code reflects CC assignment.
(PDF)

Figure S2 **Minimum spanning tree showing the relative discriminatory power of MLVA-8_Bilthoven_ and MLVA-16_Orsay_ for typing CC398.** The color code reflects the spa type. Part A (left): MST based upon the full MLVA-16_Orsay_ data. Nineteen genotypes are resolved. Part B (right): MST based upon the MLVA-8_Bilthoven_ subset of loci.
(PDF)

Figure S3 **Minimum spanning tree for the poultry isolates.** The minimum spanning tree is identical to the one shown in Figure 4 except for the color code. All human isolates are grayed, MSSA animal and food isolates are shown in green, MRSA isolates in red. Poultry isolates collecting from living animals are cross-hatched with horizontal lines. Poultry isolates from food products are cross-hatched with vertical lines. The MSSA CC45 poultry isolate was collected from a cooked chicken involved in a food poisoning event.
(PDF)

Table S1 **Isolates used in this study.**
(DOC)
