# Supplementary figures and images for: High Throughput Multiple Locus Variable Number of Tandem Repeat Analysis (MLVA) of Staphylococcus aureus from Human, Animal and Food Sources
Source: PLoS One. 2012 May 2;7(5):e33967. doi: 10.1371/journal.pone.0033967 (PMC3342327; doi:10.1371/journal.pone.0033967)

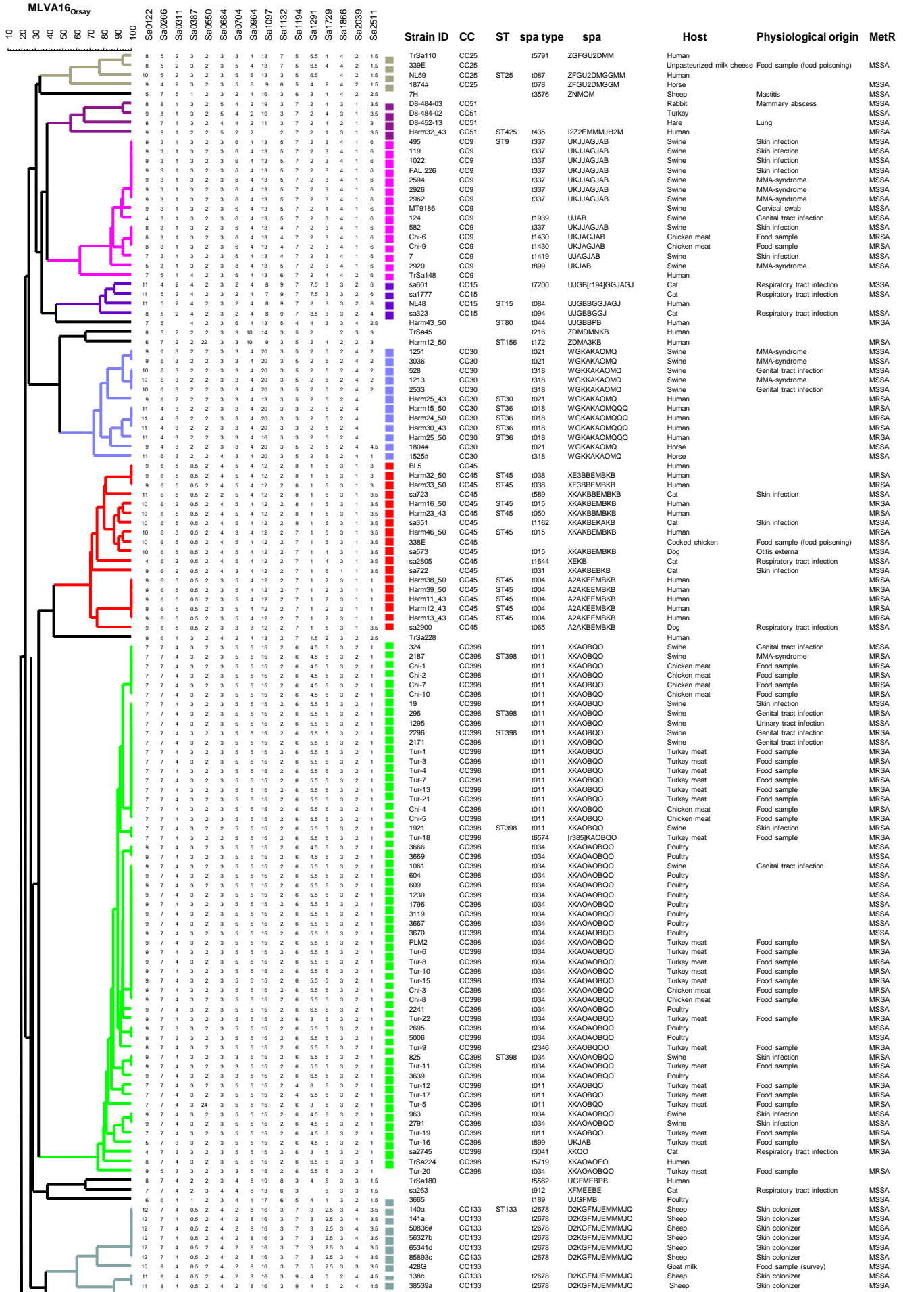

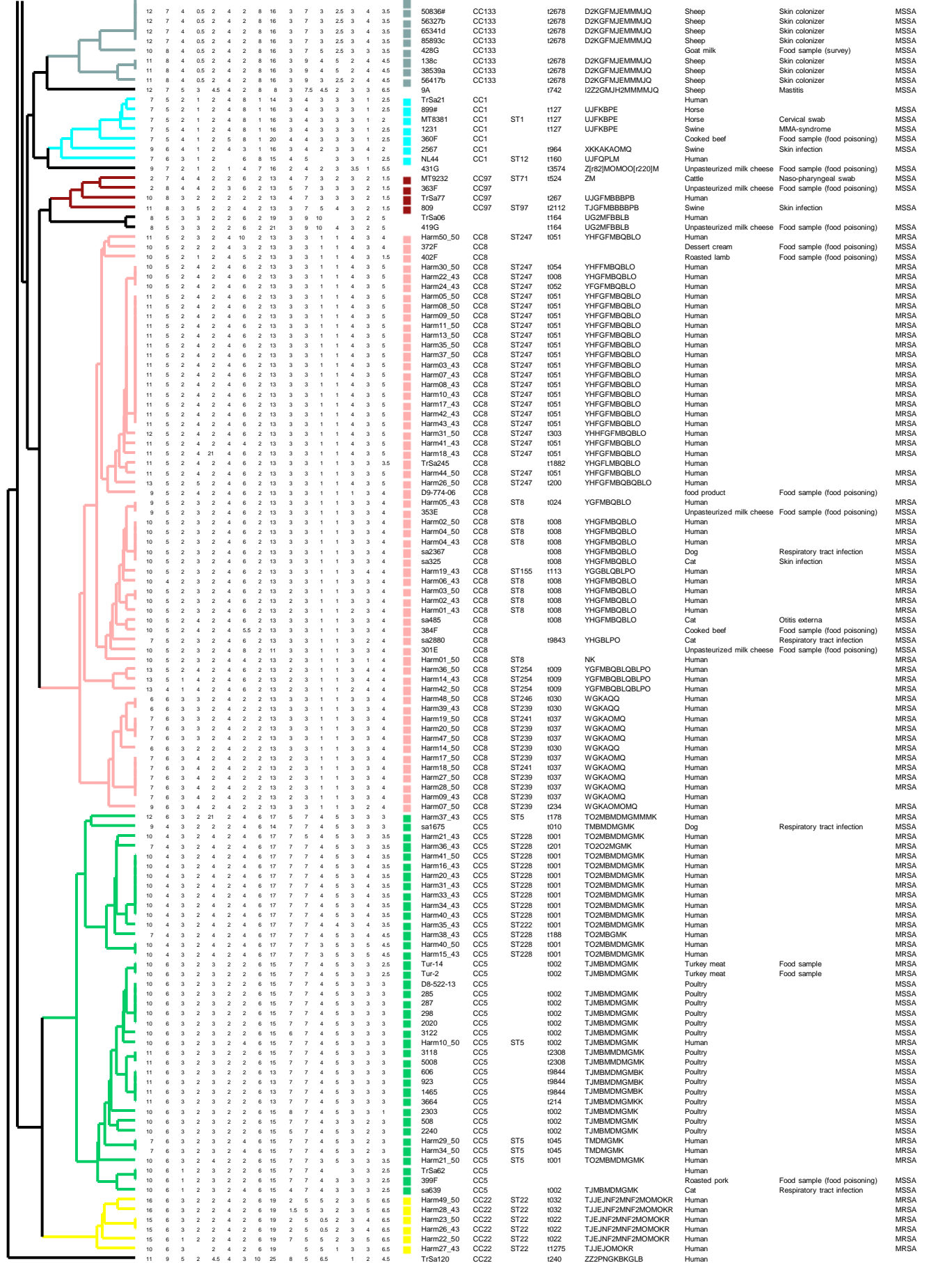

Supplement: Figure S1 — Dendrogram deduced from the clustering of the 251 S. aureus animal-associated isolates and human strains from the HARMONY collection using MLVA-16Orsay. The color code reflects CC assignment. (PDF) [file pone.0033967.s001.pdf]

A

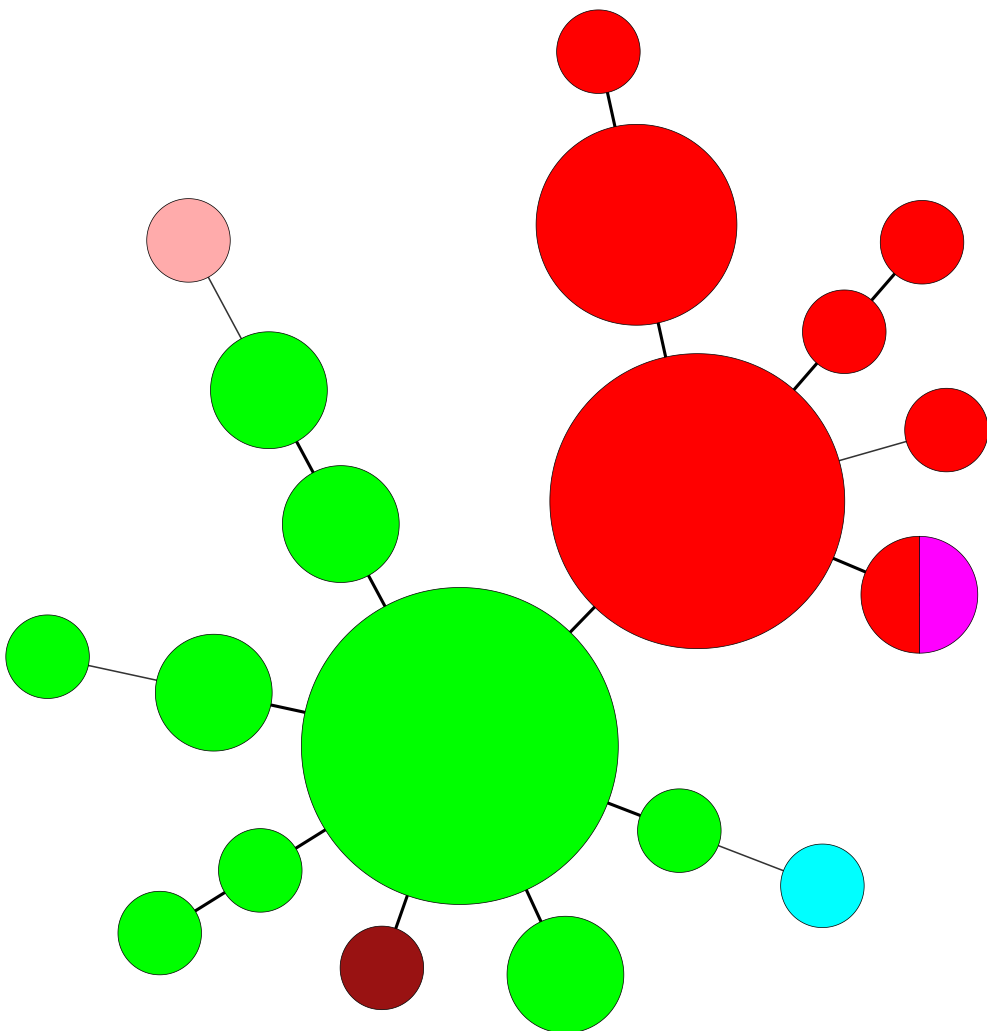

B

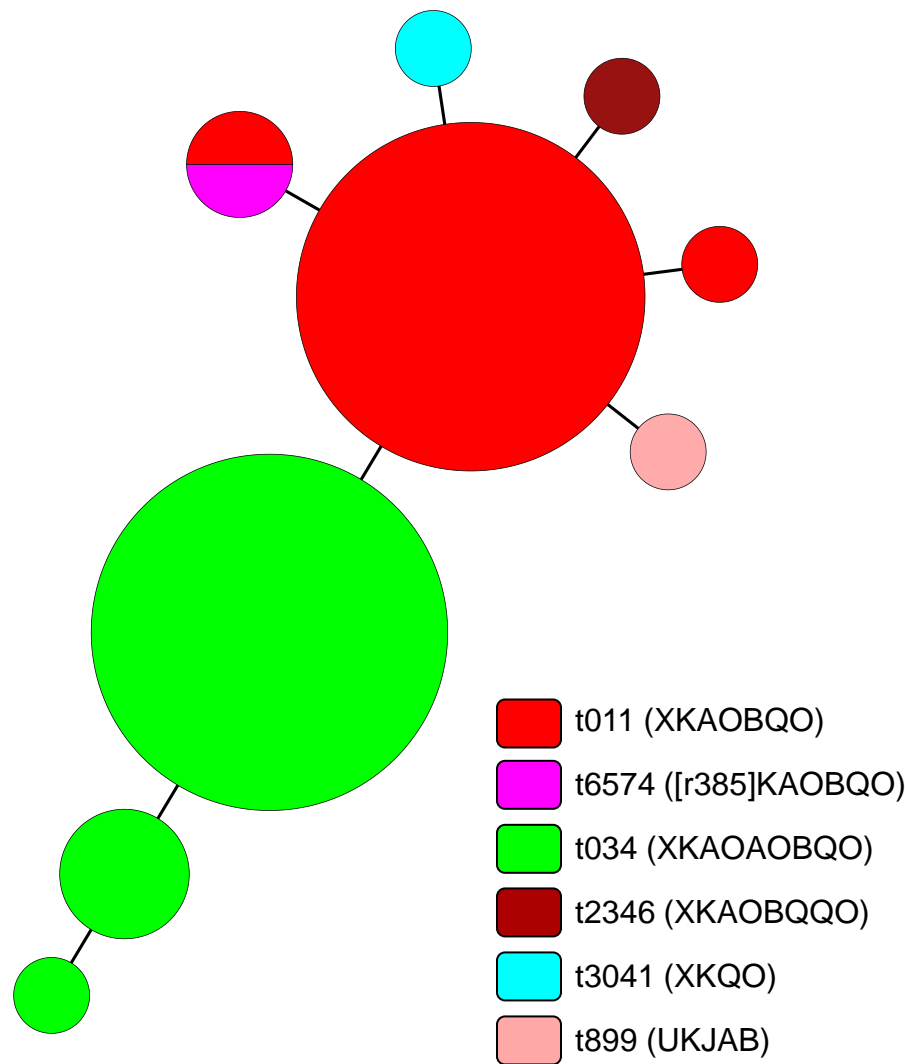

Supplement: Figure S2 — Minimum spanning tree showing the relative discriminatory power of MLVA-8Bilthoven and MLVA-16Orsay for typing CC398. The color code reflects the spa type. Part A (left): MST based upon the full MLVA-16Orsay data. Nineteen genotypes are resolved. Part B (right): MST based upon the MLVA-8Bilthoven subset of loci. (PDF) [file pone.0033967.s002.pdf]

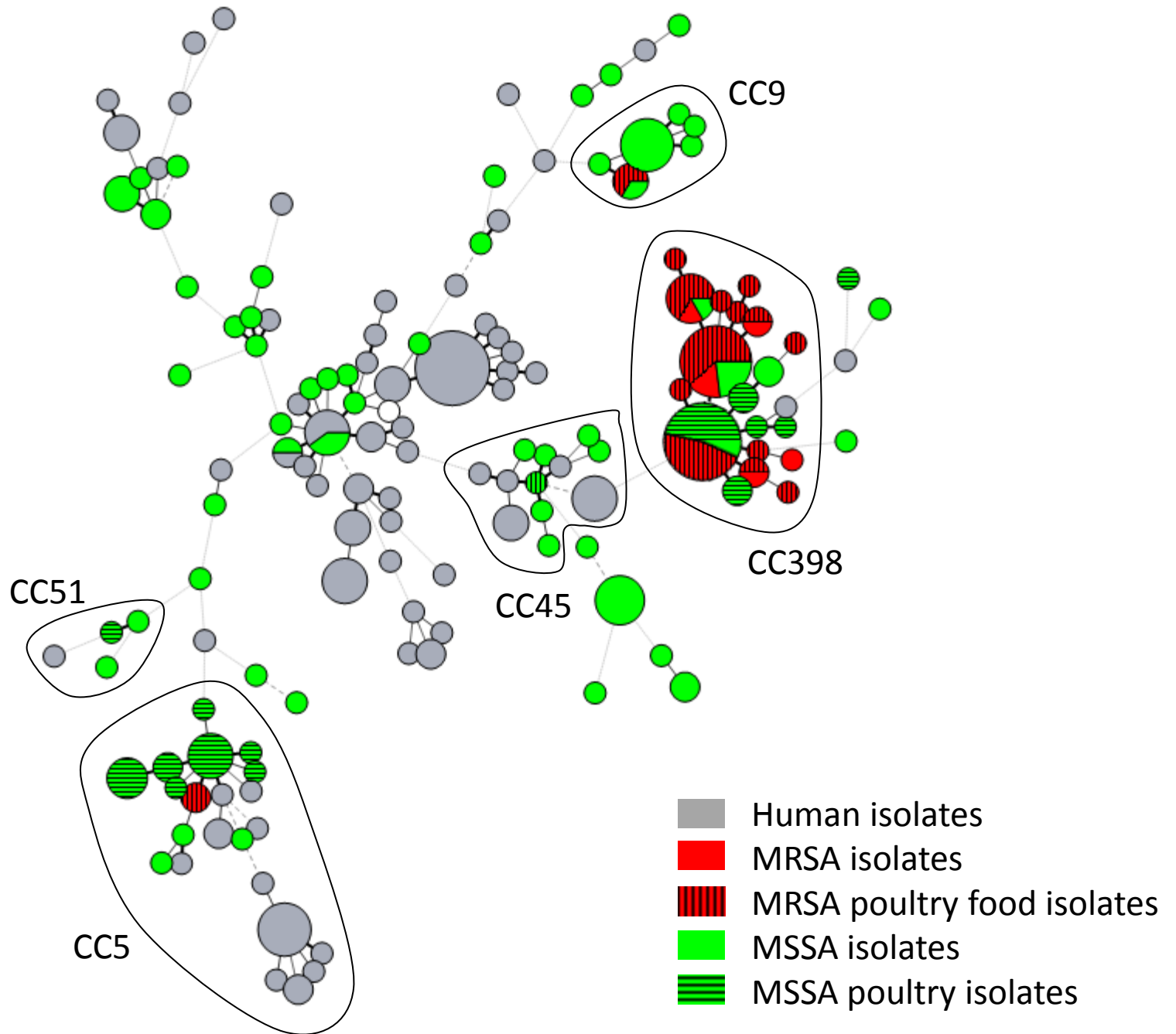

Supplement: Figure S3 — Minimum spanning tree for the poultry isolates. The minimum spanning tree is identical to the one shown in Figure 4 except for the color code. All human isolates are grayed, MSSA animal and food isolates are shown in green, MRSA isolates in red. Poultry isolates collecting from living animals are cross-hatched with horizontal lines. Poultry isolates from food products are cross-hatched with vertical lines. The MSSA CC45 poultry isolate was collected from a cooked chicken involved in a food poisoning event. (PDF) [file pone.0033967.s003.pdf]
