# Supplementary material for: High Throughput Multiple Locus Variable Number of Tandem Repeat Analysis (MLVA) of Staphylococcus aureus from Human, Animal and Food Sources
Source: PLoS One. 2012 May 2;7(5):e33967. doi: 10.1371/journal.pone.0033967 (PMC3342327; doi:10.1371/journal.pone.0033967)
Supplement: Table S1 — Isolates used in this study. (DOC) [file pone.0033967.s004.doc]

Table S1. Isolates used in this study

| **Strain ID** | **Host** | **Physiological origin** | **Year** | ***spa* type** | **Oxa** | **Origin** | **Source** |
| --- | --- | --- | --- | --- | --- | --- | --- |
| 7 | Swine | Skin infection | 2004 | t1419 | MSSA | Drewitz, Germany | S. Schwarz, FLI |
| 19 | Swine | Skin infection | 2004 | t011 | MSSA | Kissing, Germany | S. Schwarz, FLI |
| 495 | Swine | Skin infection | 2004 | t337 | MSSA | Germany | S. Schwarz, FLI |
| 582 | Swine | Skin infection | 2004 | t1430 | MSSA | Mettingen, Germany | S. Schwarz, FLI |
| 809 | Swine | Skin infection | 2004 | t2112 | MSSA | Harriehausen, Germany | S. Schwarz, FLI |
| 825 | Swine | Skin infection | 2004 | t034 | MRSA | Hodenhagen, Germany | S. Schwarz, FLI |
| 963 | Swine | Skin infection | 2004 | t034 | MSSA | Obermützkow, Germany | S. Schwarz, FLI |
| 1921 | Swine | Skin infection | 2004 | t011 | MRSA | Nuthe-Urstromtal, Germany | S. Schwarz, FLI |
| 119 | Swine | Skin infection | 2004 | t337 | MSSA | Aldersbach, Germany | S. Schwarz, FLI |
| 1022 | Swine | Skin infection | 2004 | t337 | MSSA | Solingen, Germany | S. Schwarz, FLI |
| FAL 226 | Swine | Skin infection | 2005 | t337 | MSSA | Heek, Germany | S. Schwarz, FLI |
| 2567 | Swine | Skin infection | 2005 | t964 | MSSA | Oschersleben, Germany | S. Schwarz, FLI |
| 2791 | Swine | Skin infection | 2005 | t034 | MSSA | Nemsdorf-Göhrendorf, Germany | S. Schwarz, FLI |
| 124 | Swine | Genital tract infection | 2004 | t1939 | MSSA | Mönchershofe, Germany | S. Schwarz, FLI |
| 296 | Swine | Genital tract infection | 2004 | t011 | MRSA | Kraft, Germany | S. Schwarz, FLI |
| 324 | Swine | Genital tract infection | 2004 | t011 | MSSA | Schönermark, Germany | S. Schwarz, FLI |
| 528 | Swine | Genital tract infection | 2004 | t318 | MSSA | Kalefeld, Germany | S. Schwarz, FLI |
| 1061 | Swine | Genital tract infection | 2004 | t034 | MSSA | Schnatzling, Germany | S. Schwarz, FLI |
| 1213 | Swine | MMA-syndrome | 2004 | t318 | MSSA | Bergen, Germany | S. Schwarz, FLI |
| 1231 | Swine | MMA-syndrome | 2004 | t127 | MSSA | Armsen, Gemany | S. Schwarz, FLI |
| 1251 | Swine | MMA-syndrome | 2004 | t021 | MSSA | Westen, Germany | S. Schwarz, FLI |
| 1295 | Swine | Urinary tract infection | 2004 | t011 | MSSA | Sandbeiendorf, Germany | S. Schwarz, FLI |
| 2187 | Swine | MMA-syndrome | 2005 | t011 | MRSA | Serbohlsdorf, Germany | S. Schwarz, FLI |
| 2296 | Swine | Genital tract infection | 2005 | t011 | MRSA | Kraft, Germany | S. Schwarz, FLI |
| 2594 | Swine | MMA-syndrome | 2005 | t337 | MSSA | Scheeßel, Germany | S. Schwarz, FLI |
| 2171 | Swine | Genital tract infection | 2005 | t011 | MSSA | Ertingen, Germany | S. Schwarz, FLI |
| 2533 | Swine | Genital tract infection | 2005 | t318 | MSSA | Baienfurt, Germany | S. Schwarz, FLI |
| 2920 | Swine | MMA-syndrome | 2005 | t899 | MSSA | Bad Fallingbostel, Germany | S. Schwarz, FLI |
| 2926 | Swine | MMA-syndrome | 2006 | t337 | MSSA | Reeßum, Germany | S. Schwarz, FLI |
| 2962 | Swine | MMA-syndrome | 2006 | t337 | MSSA | Oppershausen, Germany | S. Schwarz, FLI |
| 3036 | Swine | MMA-syndrome | 2006 | t021 | MSSA | Oppershausen, Germany | S. Schwarz, FLI |
| MT9186 | Swine | Cervical swab | 2009 |  | MSSA | Manche, France | M. Treilles, LDA50 |
| 285 | Chicken | Septicaemia | 2004 | t002 | MSSA | Rietberg, Germany | S. Schwarz, FLI |
| 287 | Chicken | Septicaemia | 2004 | t002 | MSSA | Delbrück, Germany | S. Schwarz, FLI |
| 298 | Chicken | Septicaemia | 2004 | t002 | MSSA | Rietberg, Germany | S. Schwarz, FLI |
| 508 | Chicken | Septicaemia | 2004 | t002 | MSSA | Mennewitz, Germany | S. Schwarz, FLI |
| 604 | Turkey | Septicaemia | 2004 | t034 | MSSA | Steinhagen, Germany | S. Schwarz, FLI |
| 606 | Chicken | Septicaemia | 2004 | t9844 | MSSA | Rheda-Wiedenbrück, Germany | S. Schwarz, FLI |
| 609 | Turkey | Septicaemia | 2004 | t034 | MSSA | Stadtlohn, Germany | S. Schwarz, FLI |
| 923 | Chicken | Septicaemia | 2004 | t9844 | MSSA | Sprachbrücken, Germany | S. Schwarz, FLI |
| 1230 | Turkey | Septicaemia | 2004 | t034 | MSSA | Ebersdorf, Germany | S. Schwarz, FLI |
| 1465 | Chicken | Septicaemia | 2004 | t9844 | MSSA | Rüssel, Germany | S. Schwarz, FLI |
| 1796 | Turkey | Septicaemia | 2004 | t034 | MSSA | Lorup, Germany | S. Schwarz, FLI |
| 2020 | Chicken | Septicaemia | 2004 | t002 | MSSA | Fladder, Germany | S. Schwarz, FLI |
| 2240 | Turkey | Septicaemia | 2004 | t002 | MSSA | Dötlingen, Germany | S. Schwarz, FLI |
| 2241 | Turkey | Septicaemia | 2004 | t034 | MSSA | Wardenburg, Germany | S. Schwarz, FLI |
| 2303 | Chicken | Septicaemia | 2004 | t002 | MSSA | Rietberg, Germany | S. Schwarz, FLI |
| 2695 | Turkey | Septicaemia | 2004 | t034 | MSSA | Wadersloh, Germany | S. Schwarz, FLI |
| 3118 | Chicken | Septicaemia | 2004 | t2308 | MSSA | Simmerhausen, Germany | S. Schwarz, FLI |
| 3119 | Turkey | Septicaemia | 2004 | t034 | MSSA | Rheda-Wiedenbrück, Germany | S. Schwarz, FLI |
| 3122 | Chicken | Septicaemia | 2004 | t002 | MSSA | Nieheim-Oeynhausen, Germany | S. Schwarz, FLI |
| 3639 | Turkey | Septicaemia | 2004 | t034 | MSSA | Recke, Germany | S. Schwarz, FLI |
| 3664 | Chicken | Septicaemia | 2004 | t214 | MSSA | Ostbevern, Germany | S. Schwarz, FLI |
| 3665 | Turkey | Septicaemia | 2004 | t189 | MSSA | Reken Großreken, Germany | S. Schwarz, FLI |
| 3666 | Turkey | Septicaemia | 2004 | t034 | MSSA | Steinhagen, Germany | S. Schwarz, FLI |
| 3667 | Turkey | Septicaemia | 2004 | t034 | MSSA | Rheda-Wiedenbrück, Germany | S. Schwarz, FLI |
| 3669 | Turkey | Septicaemia | 2004 | t034 | MSSA | Steinhagen, Germany | S. Schwarz, FLI |
| 3670 | Turkey | Septicaemia | 2004 | t034 | MSSA | Rheda-Wiedenbrück, Germany | S. Schwarz, FLI |
| 5006 | Turkey | Septicaemia | 2004 | t034 | MSSA | Verl, Germany | S. Schwarz, FLI |
| 5008 | Chicken | Septicaemia | 2004 | t2308 | MSSA | Barnstedt, Germany | S. Schwarz, FLI |
| D8-522-13 | Poultry | Unknown | 1972 |  | MSSA | Unknown | F. Gilbert, INRA |
| D8-484-02 | Turkey | Unknown | 1989 |  | MSSA | Unknown | F. Gilbert, INRA |
| 138c | Sheep | Skin colonizer | 2010 | t2678 | MSSA | Neustadt, Germany | S. Schwarz, FLI |
| 140a | Sheep | Skin colonizer | 2010 | t2678 | MSSA | Neustadt, Germany | S. Schwarz, FLI |
| 141a | Sheep | Skin colonizer | 2010 | t2678 | MSSA | Neustadt, Germany | S. Schwarz, FLI |
| 38539a | Sheep | Skin colonizer | 2010 | t2678 | MSSA | Neustadt, Germany | S. Schwarz, FLI |
| 50836# | Sheep | Skin colonizer | 2010 | t2678 | MSSA | Neustadt, Germany | S. Schwarz, FLI |
| 56327b | Sheep | Skin colonizer | 2010 | t2678 | MSSA | Neustadt, Germany | S. Schwarz, FLI |
| 56417b | Sheep | Skin colonizer | 2010 | t2678 | MSSA | Neustadt, Germany | S. Schwarz, FLI |
| 65341d | Sheep | Skin colonizer | 2010 | t2678 | MSSA | Neustadt, Germany | S. Schwarz, FLI |
| 85893c | Sheep | Skin colonizer | 2010 | t2678 | MSSA | Neustadt, Germany | S. Schwarz, FLI |
| 9A | Sheep | Mastitis | 1995 |  | MSSA | Pyrénées-Atlantiques, France | D. Bergonier, INRA-ENVT |
| 7H | Sheep | Mastitis | 1996 |  | MSSA | Pyrénées-Atlantiques, France | D. Bergonier, INRA-ENVT |
| sa263 | Cat | Respiratory tract infection | 2004 | t912 | MSSA | Salzgitter, Germany | S. Schwarz, FLI |
| sa323 | Cat | Respiratory tract infection | 2004 | t094 | MSSA | Borkwalde, Germany | S. Schwarz, FLI |
| sa325 | Cat | Skin infection | 2004 | t008 | MSSA | Niedergörsdorf, Germany | S. Schwarz, FLI |
| sa351 | Cat | Skin infection | 2004 | t1162 | MSSA | Berlin, Germany | S. Schwarz, FLI |
| sa485 | Cat | Otitis externa | 2004 | t008 | MSSA | München, Germany | S. Schwarz, FLI |
| sa601 | Cat | Respiratory tract infection | 2004 | t7200 | MSSA | Berlin, Germany | S. Schwarz, FLI |
| sa639 | Cat | Respiratory tract infection | 2004 | t002 | MSSA | Berlin, Germany | S. Schwarz, FLI |
| sa722 | Cat | Skin infection | 2004 | t031 | MSSA | Loonig, Germany | S. Schwarz, FLI |
| sa723 | Cat | Skin infection | 2004 | t589 | MSSA | Hamburg, Germany | S. Schwarz, FLI |
| sa1777 | Cat | Respiratory tract infection | 2004 |  | MSSA | Hamburg, Germany | S. Schwarz, FLI |
| sa2745 | Cat | Respiratory tract infection | 2005 | t3041 | MRSA | Berlin, Germany | S. Schwarz, FLI |
| sa2805 | Cat | Respiratory tract infection | 2005 | t1644 | MSSA | Leitzkau, Germany | S. Schwarz, FLI |
| sa2880 | Cat | Respiratory tract infection | 2008 | t9843 | MSSA | Geesthacht, Germany | S. Schwarz, FLI |
| 899# | Horse | Unknown | 1992 | t127 | MSSA | Germany | S. Schwarz, FLI |
| 1525# | Horse | Unknown | 1992 | t318 | MSSA | Germany | S. Schwarz, FLI |
| 1804# | Horse | Unknown | 1992 | t021 | MSSA | Germany | S. Schwarz, FLI |
| 1874# | Horse | Unknown | 1992 | t078 | MSSA | Germany | S. Schwarz, FLI |
| MT8381 | Horse | Cervical swab | 2008 |  | MSSA | Manche, France | M. Treilles, LDA50 |
| sa573 | Dog | Otitis externa | 2004 | t015 | MSSA | 34329 Nieste, Germany | S. Schwarz, FLI |
| sa1675 | Dog | Respiratory tract infection | 2004 | t010 | MSSA | 22850 Norderstedt, Germany | S. Schwarz, FLI |
| sa2367 | Dog | Respiratory tract infection | 2005 | t008 | MSSA | 22523 Hamburg, Germany | S. Schwarz, FLI |
| sa2900 | Dog | Respiratory tract infection | 2005 | t065 | MSSA | 22523 Hamburg, Germany | S. Schwarz, FLI |
| MT9232 | Cattle | Naso-pharyngeal swab | 2009 |  | MSSA | Manche, France | M. Treilles, LDA50 |
| D8-452-13 | Hare | Lung | 1988 |  | MSSA | Puy de Dôme, France | F. Gilbert, INRA |
| D8-484-03 | Rabbit | Mammary abscess | 1989 |  | MSSA | Vendée, France | F. Gilbert, INRA |
| PLM2 | Turkey meat | Food sample | 2009 | t034 | MRSA | Rhineland-Palatinate, Germany | S. Schwarz, FLI |
| Tur-1 | Turkey meat | Food sample | 2009 | t002 | MRSA | Rhineland-Palatinate, Germany | S. Schwarz, FLI |
| Tur-2 | Turkey meat | Food sample | 2009 | t011 | MRSA | Rhineland-Palatinate, Germany | S. Schwarz, FLI |
| Tur-3 | Turkey meat | Food sample | 2009 | t011 | MRSA | Rhineland-Palatinate, Germany | S. Schwarz, FLI |
| Tur-4 | Turkey meat | Food sample | 2009 | t011 | MRSA | Rhineland-Palatinate, Germany | S. Schwarz, FLI |
| Tur-5 | Turkey meat | Food sample | 2009 | t011 | MRSA | Rhineland-Palatinate, Germany | S. Schwarz, FLI |
| Tur-6 | Turkey meat | Food sample | 2009 | t034 | MRSA | Rhineland-Palatinate, Germany | S. Schwarz, FLI |
| Tur-7 | Turkey meat | Food sample | 2009 | t011 | MRSA | Rhineland-Palatinate, Germany | S. Schwarz, FLI |
| Tur-8 | Turkey meat | Food sample | 2009 | t034 | MRSA | Rhineland-Palatinate, Germany | S. Schwarz, FLI |
| Tur-9 | Turkey meat | Food sample | 2009 | t2346 | MRSA | Rhineland-Palatinate, Germany | S. Schwarz, FLI |
| Tur-10 | Turkey meat | Food sample | 2009 | t034 | MRSA | Rhineland-Palatinate, Germany | S. Schwarz, FLI |
| Tur-11 | Turkey meat | Food sample | 2009 | t034 | MRSA | Rhineland-Palatinate, Germany | S. Schwarz, FLI |
| Tur-12 | Turkey meat | Food sample | 2009 | t011 | MRSA | Rhineland-Palatinate, Germany | S. Schwarz, FLI |
| Tur-13 | Turkey meat | Food sample | 2009 | t011 | MRSA | Rhineland-Palatinate, Germany | S. Schwarz, FLI |
| Tur-14 | Turkey meat | Food sample | 2009 | t002 | MRSA | Rhineland-Palatinate, Germany | S. Schwarz, FLI |
| Tur-15 | Turkey meat | Food sample | 2009 | t034 | MRSA | Rhineland-Palatinate, Germany | S. Schwarz, FLI |
| Tur-16 | Turkey meat | Food sample | 2009 | t899 | MRSA | Rhineland-Palatinate, Germany | S. Schwarz, FLI |
| Tur-17 | Turkey meat | Food sample | 2009 | t011 | MRSA | Rhineland-Palatinate, Germany | S. Schwarz, FLI |
| Tur-18 | Turkey meat | Food sample | 2009 | t6574 | MRSA | Rhineland-Palatinate, Germany | S. Schwarz, FLI |
| Tur-19 | Turkey meat | Food sample | 2009 | t011 | MRSA | Rhineland-Palatinate, Germany | S. Schwarz, FLI |
| Tur-20 | Turkey meat | Food sample | 2009 | t034 | MRSA | Rhineland-Palatinate, Germany | S. Schwarz, FLI |
| Tur-21 | Turkey meat | Food sample | 2009 | t011 | MRSA | Rhineland-Palatinate, Germany | S. Schwarz, FLI |
| Tur-22 | Turkey meat | Food sample | 2009 | t034 | MRSA | Rhineland-Palatinate, Germany | S. Schwarz, FLI |
| Chi-1 | Chicken meat | Food sample | 2009 | t011 | MRSA | Rhineland-Palatinate, Germany | S. Schwarz, FLI |
| Chi-2 | Chicken meat | Food sample | 2009 | t011 | MRSA | Rhineland-Palatinate, Germany | S. Schwarz, FLI |
| Chi-3 | Chicken meat | Food sample | 2009 | t034 | MRSA | Rhineland-Palatinate, Germany | S. Schwarz, FLI |
| Chi-4 | Chicken meat | Food sample | 2009 | t011 | MRSA | Rhineland-Palatinate, Germany | S. Schwarz, FLI |
| Chi-5 | Chicken meat | Food sample | 2009 | t011 | MRSA | Rhineland-Palatinate, Germany | S. Schwarz, FLI |
| Chi-6 | Chicken meat | Food sample | 2009 | t1430 | MRSA | Rhineland-Palatinate, Germany | S. Schwarz, FLI |
| Chi-7 | Chicken meat | Food sample | 2009 | t011 | MRSA | Rhineland-Palatinate, Germany | S. Schwarz, FLI |
| Chi-8 | Chicken meat | Food sample | 2009 | t034 | MRSA | Rhineland-Palatinate, Germany | S. Schwarz, FLI |
| Chi-9 | Chicken meat | Food sample | 2009 | t1430 | MRSA | Rhineland-Palatinate, Germany | S. Schwarz, FLI |
| Chi-10 | Chicken meat | Food sample | 2009 | t011 | MRSA | Rhineland-Palatinate, Germany | S. Schwarz, FLI |
| 428G | Goat milk | Food sample | 2002 |  | MSSA | Saône et Loire, France | M.-L. De Buyser, ANSES |
| 338E | Cooked chicken | Food sample (food poisoning) | 1989 |  | MSSA | Paris, France | M.-L. De Buyser, ANSES |
| 419G | Sheep UMC* | Food sample (food poisoning) | 2001 |  | MSSA | Puy de Dôme, France | M.-L. De Buyser, ANSES |
| 301E | Sheep UMC | Food sample (food poisoning) | 1997 |  | MSSA | Aveyron, France | M.-L. De Buyser, ANSES |
| 353E | Sheep UMC | Food sample (food poisoning) | 1981 |  | MSSA | Pyrénées-Atlantiques, France | M.-L. De Buyser, ANSES |
| 339E | Sheep UMC | Food sample (food poisoning) | 1986 |  | MSSA | Landes, France | M.-L. De Buyser, ANSES |
| 363F | Sheep UMC | Food sample (food poisoning) | 1998 |  | MSSA | Cantal, France | M.-L. De Buyser, ANSES |
| 431G | Sheep UMC | Food sample (food poisoning) | 2002 |  | MSSA | Pyrénées-Atlantiques, France | M.-L. De Buyser, ANSES |
| 372F | Dessert cream | Food sample (food poisoning) | 2001 |  | MSSA | Val de Marne, France | M.-L. De Buyser, ANSES |
| 360F | Cooked beef | Food sample (food poisoning) | 1983 |  | MSSA | Oise, France | M.-L. De Buyser, ANSES |
| 384F | Cooked beef | Food sample (food poisoning) | 1983 |  | MSSA | Doubs, France | M.-L. De Buyser, ANSES |
| 399F | Roasted pork | Food sample (food poisoning) | 2001 |  | MSSA | Ille et Vilaine, France | M.-L. De Buyser, ANSES |
| 402F | Roasted lamb | Food sample (food poisoning) | 2001 |  | MSSA | Gironde, France | M.-L. De Buyser, ANSES |
| D9-774-06 |  | Food sample (food poisoning) | 1992 |  | ND | USA | F. Gilbert, INRA |

*UMC: Unpasteurized milk cheese
